# Persistent Supraventricular Tachycardia Following Central Venous Catheter Insertion: When Withdrawal of the Mechanical Stimulus Is Not Enough

**DOI:** 10.7759/cureus.117565

**Published:** 2026-10-06

**Authors:** Sanjay Bhagoutie, Amanda C Sang, Keevan Singh, Candice Ramdin, Shenelle N Balliram

**Affiliations:** 1 Anaesthesia and Intensive Care Unit, San Fernando General Hospital, San Fernando, TTO; 2 Anaesthesia and Intensive Care Unit, Department of Clinical Surgical Sciences, University of the West Indies, San Fernando, TTO

**Keywords:** catheter-associated arrhythmia, central venous catheter complications, central venous catheterization, diabetic ketoacidosis (dka), narrow-complex tachycardia

## Abstract

Cardiac arrhythmias are a recognised complication of central venous catheterisation and are usually attributed to mechanical irritation of the endocardium by the guidewire or catheter tip. These arrhythmias typically resolve following withdrawal of the mechanical stimulus. We report a 42-year-old man with heart failure with reduced ejection fraction and diabetic ketoacidosis who developed an abrupt regular narrow-complex tachycardia at approximately 217 beats/min immediately following advancement of a right internal jugular central venous catheter. The arrhythmia was associated with transient hypotension and dizziness and persisted despite removal of both the guidewire and catheter. This case illustrates that catheter manipulation may trigger a sustained supraventricular tachycardia that persists after removal of the mechanical stimulus, particularly in a patient with structural heart disease and metabolic and electrolyte abnormalities. Persistent tachyarrhythmia following catheter withdrawal should prompt assessment of haemodynamic stability and guideline-directed arrhythmia management rather than an assumption of spontaneous resolution.

## Introduction

Central venous catheter (CVC) insertion is a fundamental procedure in critical care, anaesthesia, and emergency medicine, providing reliable vascular access for administration of chemically irritant drugs, vasoactive medications, and haemodynamic monitoring. Although generally safe, CVC placement is associated with both minor and major complications, including bleeding, pneumothorax, vascular injury, infection, cardiac placement malposition and failure and arrhythmia [1,2].

Arrhythmias during CVC insertion are well documented and are most attributed to mechanical irritation of the endocardium by the guidewire or catheter tip, particularly when advanced into the right atrium or ventricle. These arrhythmias are typically transient and resolve promptly with withdrawal of the guidewire or repositioning of the catheter [3-5].

The literature is comparatively thin on cases where a supraventricular tachyarrhythmia persists after the mechanical stimulus has been removed. Most published accounts frame catheter position and arrhythmia as directly and immediately coupled - remove the trigger, the rhythm reverts.

We present a case of persistent supraventricular tachyarrhythmia following internal jugular CVC insertion that persisted despite catheter removal and required pharmacologic intervention.

## Case presentation

We report a 42-year-old male admitted to the Intensive Care Unit (ICU) of a tertiary care hospital in Trinidad with diabetic ketoacidosis (DKA) secondary to an abdominal wall abscess. His past medical history included type 2 diabetes mellitus, heart failure with a reduced ejection fraction (HFrEF) (estimated left ventricular systolic ejection fraction of approximately 40%), and a previous cerebrovascular event with residual left hemiparesis. His baseline Modified Rankin Score was 4 [6].

On admission, laboratory investigations demonstrated mild hypokalaemia, with a serum potassium concentration of 3.1 mmol/L. Serum sodium was 141 mmol/L, chloride was 108 mmol/L, and serum creatinine was 0.4 mg/dL (Table 1). Arterial blood gas analysis demonstrated a high-anion-gap metabolic acidosis with appropriate respiratory compensation (pH 7.20, PaCO₂ 21.2 mmHg, HCO₃⁻ 8.3 mmol/L and base excess −20 mmol/L) (Table 1). The calculated anion gap was 24.7 mmol/L, consistent with his diagnosis of DKA. Despite the metabolic derangement and hypokalaemia, the admission electrocardiogram (ECG) demonstrated sinus rhythm without significant abnormalities.

**Table 1:** Electrolyte and ABG values at admission and at the time of SVT ABG: Arterial blood gas; SVT: supraventricular tachycardia

Parameter	Admission	At SVT onset	Reference range
Sodium	141	137	135-145 mmol/L
Potassium	3.1	2.7	3.5-5.1 mmol/L
Chloride	108	102	98-107 mmol/L
Creatinine	0.4	0.6	0.7-1.2 mg/dL
Corrected calcium	Unavailable	Unavailable	8.5-10.5 mg/dL
Magnesium	Unavailable	Unavailable	0.65-1.05 mmol/L
Phosphate	Unavailable	Unavailable	1.8-2.3 mmol/L
pH	7.2	----	7.35-7.45
PaCO_2_	21.2	----	35-45 mmHg
PaO_2_	Unavailable	----	75-100 mmHg
HCO_3 _^-^	8.3	----	22-26 mEq/L
Base excess	-20	----	-3 - 3mmol/L
Anion gap	24.7	----	8-12 mEq/L

Source control was achieved by incision and drainage of the abscess under general anaesthesia, and he was then admitted to the ICU for ongoing management of DKA. The patient remained hemodynamically stable but required monitoring and intravenous therapies, including fluid resuscitation, insulin infusion, empirical antibiotic therapy and potassium replacement for hypokalaemia. Despite dilution, peripheral potassium administration caused severe burning pain at the infusion site, prompting the decision to obtain central venous access for ongoing electrolyte replacement and reliable vascular access.

Under continuous electrocardiogram (ECG) monitoring and ultrasound guidance, the right internal jugular vein was cannulated using the Seldinger technique. Venous puncture was achieved on first attempt. The 50-cm guidewire lacked depth graduations; therefore, the precise insertion depth could not be determined. The guidewire was advanced without resistance, and no ectopy or other ECG changes were observed during guidewire advancement or tract dilatation. Following tract dilatation, a 15cm, 7 French, triple-lumen CVC was placed.

Immediately, with the advancement of the catheter to the 15cm mark, over the guidewire, the patient developed an abrupt narrow-complex tachyarrhythmia associated with dizziness. Vital signs revealed a heart rate of approximately 217 beats per minute on telemetry, blood pressure of 72/53, oxygen saturation 100% on room air and GCS of 15. The hypotension resolved within approximately 30 seconds, with repeat blood pressure of 103/59 mmHg, despite persistence of the tachycardia.

Although the initial hypotension accompanied by dizziness raised concern for haemodynamic instability and would ordinarily prompt consideration of synchronized cardioversion, the hypotension was transient. The patient remained conscious, without chest pain, dyspnoea or hypoxaemia. The guidewire was removed first and then the catheter was progressively withdrawn and ultimately removed. However, despite removal of CVC, the tachyarrhythmia persisted.

Point-of-care lung ultrasonography demonstrated no evidence of pneumothorax. Vagal manoeuvres were attempted and unsuccessful. A 12-lead electrocardiogram demonstrated a regular narrow-complex tachycardia at approximately 212 beats/min, with a QRS duration of approximately 94 ms and no clearly discernible preceding P waves (Figure 1).

**Figure 1:**
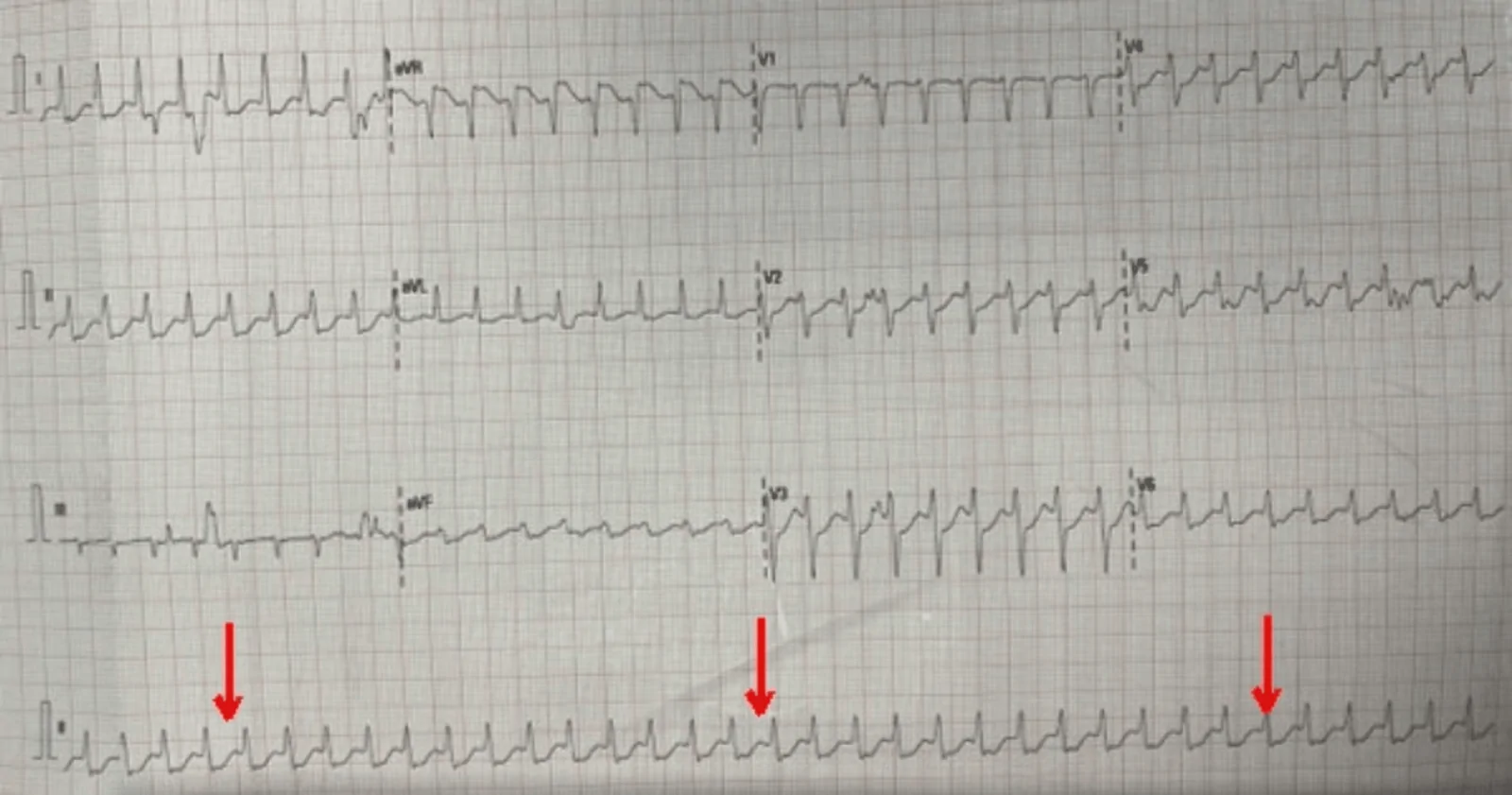
Twelve-lead electrocardiogram demonstrating persistent regular narrow-complex supraventricular tachycardia following removal of the central venous catheter

As haemodynamic stability had been restored, pharmacological treatment was pursued with continuous haemodynamic monitoring and synchronized cardioversion immediately available should instability recur. Initial pharmacological management consisted of intravenous amiodarone 150 mg, selected at the bedside because the precise tachyarrhythmia mechanism had not yet been established and the patient had underlying structural heart disease. Following review of the 12-lead ECG demonstrating a regular narrow-complex tachycardia, intravenous adenosine 6 mg was administered approximately seven minutes after arrhythmia onset, resulting in abrupt termination and restoration of sinus rhythm at approximately 120 beats/min. 

The regular narrow-complex morphology and subsequent abrupt termination with adenosine were suggestive of an atrioventricular node-dependent re-entrant tachycardia, such as atrioventricular nodal re-entrant tachycardia or orthodromic atrioventricular re-entrant tachycardia; however, the precise electrophysiological mechanism could not be established from the available ECG, see Figure 1.

A post-procedural chest radiograph showed no acute abnormality. The patient continued to be haemodynamically stable after rhythm conversion, with no recurrence of sustained supraventricular tachycardia (SVT) during the remainder of his ICU admission.

## Discussion

Cardiac arrhythmias during central venous catheterisation are a well-recognised complication, most commonly resulting from over-insertion of the guidewire or catheter contact with the endocardial surface, causing mechanical irritation of the endocardium [3-5]. One RCT found that the highest incidence of arrhythmic episodes occurred when the guidewire was inserted to a depth beyond 20 cm [4].

Reported incidence varies according to catheter type, insertion technique, monitoring and definition of arrhythmia; one study reported an incidence of 1.6% [5]. The types of arrhythmias documented include most commonly atrial arrhythmias and ventricular ectopy [4]. These arrhythmias are typically transient and resolve with withdrawal of the offending stimulus [4,5].

The close temporal relationship between catheter advancement and abrupt arrhythmia onset supports catheter manipulation as the precipitating event in this case, although a direct causal mechanism cannot be proven. The absence of rhythm disturbance during guidewire advancement and the onset immediately after catheter advancement are particularly relevant. Mechanical stimulation may have initiated a re-entrant tachycardia that subsequently became self-sustaining after the mechanical stimulus had been removed. Similar sustained supraventricular tachyarrhythmias associated with CVC placement have been described, although they remain uncommon [7-10].

Despite the metabolic derangement and hypokalaemia, the admission ECG demonstrated sinus rhythm without significant abnormalities. At the time of the arrhythmia, serum potassium had decreased to 2.7 mmol/L. Additional electrolyte abnormalities that may have contributed to myocardial excitability could not be excluded due to ongoing laboratory reagent limitations. At the time of the event, the patient was normothermic with a core temperature of approximately 36°C and had a normal haemoglobin concentration. He was not receiving vasopressor or inotropic support, and no recognised QT-prolonging medications were being administered. There was no documented previous history of cardiac arrhythmia.

Several patient-specific factors may have increased susceptibility to sustained arrhythmia. HFrEF is associated with structural and electrical myocardial remodelling that predisposes to arrhythmogenesis [11]. DKA may further increase arrhythmic risk through electrolyte disturbances such as hypokalemia and acid-base disturbances [12,13]. In this patient, potassium decreased from 3.1 mmol/L on admission to 2.7 mmol/L at the time of the event (Table 1). These factors likely created a susceptible electrophysiological substrate upon which catheter manipulation acted as the immediate trigger for the tachyarrhythmia [12,14].

During SVT, it is essential to assess for signs of haemodynamic instability. These signs include angina, hypotension, altered mental status, hypoxia, and evidence of poor end-organ perfusion or shock. Early consideration for synchronised electrical cardioversion for unstable SVT remains the first-line treatment. In our case, the period of hypotension was transient and thus pharmacotherapy was utilised [15].

Amiodarone, although commonly employed for various arrhythmias, is not regarded as the primary treatment for acute SVT, especially in cases of re-entrant tachycardias involving the atrioventricular node. Its relatively slower onset of action may account for its limited efficacy in this acute setting [15]. Adenosine swiftly and transiently inhibits atrioventricular nodal conduction, rendering it more efficacious in stopping re-entrant SVTs [15].

Published literature predominantly emphasises withdrawal or repositioning of the guidewire or catheter when catheter-associated arrhythmia occurs; comparatively little guidance addresses management when the arrhythmia persists after complete removal of the mechanical stimulus [2,5].

Although intracardiac guidewire position was not demonstrated, limiting guidewire advancement is an important strategy for reducing mechanically induced arrhythmias [2,4]. Ultrasound guidance improves the safety of venous access but does not, by itself, confirm final catheter-tip position [2]. Where available, adjuncts such as intracavitary ECG, echocardiographic visualisation of the superior vena cava-right atrial region, agitated-saline or microbubble techniques, and post-procedural radiography may assist with tip localisation or complication assessment [16].

## Conclusions

Persistent supraventricular tachyarrhythmia following CVC insertion is an uncommon but clinically important complication. Although catheter- and guidewire-induced arrhythmias are typically transient, this case demonstrates that a tachyarrhythmia temporarily associated with catheter manipulation may persist despite removal of the mechanical stimulus, particularly in a patient with underlying structural heart disease and metabolic or electrolyte disturbances.

Persistent tachycardia following catheter withdrawal should therefore prompt immediate assessment of haemodynamic stability, correction of reversible precipitating factors, and guideline-directed arrhythmia management rather than an assumption of spontaneous resolution. Careful attention to guidewire insertion depth, continuous rhythm monitoring and appropriate confirmation of catheter tip position remain important measures for reducing catheter-associated complications.
